# Water-Soluble Phthalocyanines Selectively Bind to Albumin Dimers: A Green Approach Toward Enhancing Tumor-Targeted Photodynamic Therapy

**DOI:** 10.7150/thno.35210

**Published:** 2019-08-14

**Authors:** Xingshu Li, Keunsoo Jeong, Yoonji Lee, Tian Guo, Dayoung Lee, Jeongmin Park, Nahyun Kwon, Jung-Hyun Na, Seung Kon Hong, Sun-Shin Cha, Jian-Dong Huang, Sun Choi, Sehoon Kim, Juyoung Yoon

**Affiliations:** 1 College of Chemistry, State Key Laboratory of Photocatalysis on Energy and Environment, Fujian Provincial Key Laboratory of Cancer Metastasis Chemoprevention and Chemotherapy, Fuzhou University, Fuzhou 350108, China.; 2 Department of Chemistry and Nano Science, Ewha Womans University, Seoul 03760, Republic of Korea.; 3 Center for Theragnosis, Korea Institute of Science and Technology (KIST), Seoul 02792, Republic of Korea.; 4 College of Pharmacy and Graduate School of Pharmaceutical Sciences, Ewha Womans University, Seoul 03760, Republic of Korea.; 5 KU-KIST Graduate School of Converging Science and Technology, Korea University, Seoul 02841, Republic of Korea.

**Keywords:** photodynamic therapy, phthalocyanine, albumin dimer, natural carrier, tumor targeting

## Abstract

Targeted delivery of therapeutic agents is of particular interest in the field of cancer treatment. However, there is an urgent need for developing clinically promising targeting approaches that can be readily administered in a green manner.

**Methods**: Five phthalocyanine derivatives bearing different anionic and cationic groups were designed and synthesized. Then, their binding affinity with albumin were studied using gel assays, optical spectra and computational simulation. Finally, *in vitro* and *in vivo* fluorescence imaging and photodynamic therapy (PDT) evaluations were carried out.

**Results**: The two positively charged compounds could selectively bind to albumin dimer over albumin monomer, while the three negatively charged phthalocyanines could bind to both albumin monomer and dimer. Following systemic administration, the phthalocyanines show improved tumor accumulation via transport by natural albumin. PDT evaluations indicate that one of the positively charged compounds, ZnPcN_4_, shows outstanding phototherapeutic efficacy against tumors in preclinical models.

**Conclusion**: Our findings demonstrate that the use of water-soluble phthalocyanines as photosensitizers and *in vivo* albumin as a natural carrier may provide a green and efficient approach for tumor-targeted imaging and therapy.

## Introduction

Targeted therapy, which delivers the right therapeutic agents to the right target to efficiently achieve localized control of the tumor with minimal side effects, is a highly promising cancer therapeutic strategy 1-5. However, there is an urgent need to develop clinically relevant targeting approaches that can be readily administered in a green manner.

Albumin, the most abundant protein in the blood, is closely related to the regulation of plasma colloid osmotic pressure and the transport of numerous endogenous and exogenous compounds 6,7. Many studies have claimed that albumin is most likely involved in the provision of nutrition to tumors 8. Albumin receptors that are overexpressed on cancer cells, such as glycoprotein 60 and the albumin-binding protein SPARC (secreted protein acidic and rich in cysteine), have been found to facilitate the accumulation, distribution and degradation of albumin in tumor tissues 6,9,10. Owing to its potential for enhanced permeability and retention (EPR) effect, albumin has been extensively explored as a versatile nanocarrier for drug delivery via covalent conjugation and noncovalent interactions 6,7,11-16. However, most of the existing albumin-based delivery systems utilize *in vitro* albumins and even require extra materials to construct and stabilize these systems. These albumin-based platforms suffer from certain shortcomings, including tedious fabrication, poor reproducibility, the potential immunogenicity of *in vitro* albumin and the potential toxicity of system complexes, which hamper their future clinical translation. In contrast, we herein demonstrate a green tumor-targeting approach using *in vivo* albumin as a carrier for delivering molecular dye-based photosensitizers.

**Figure 1:**
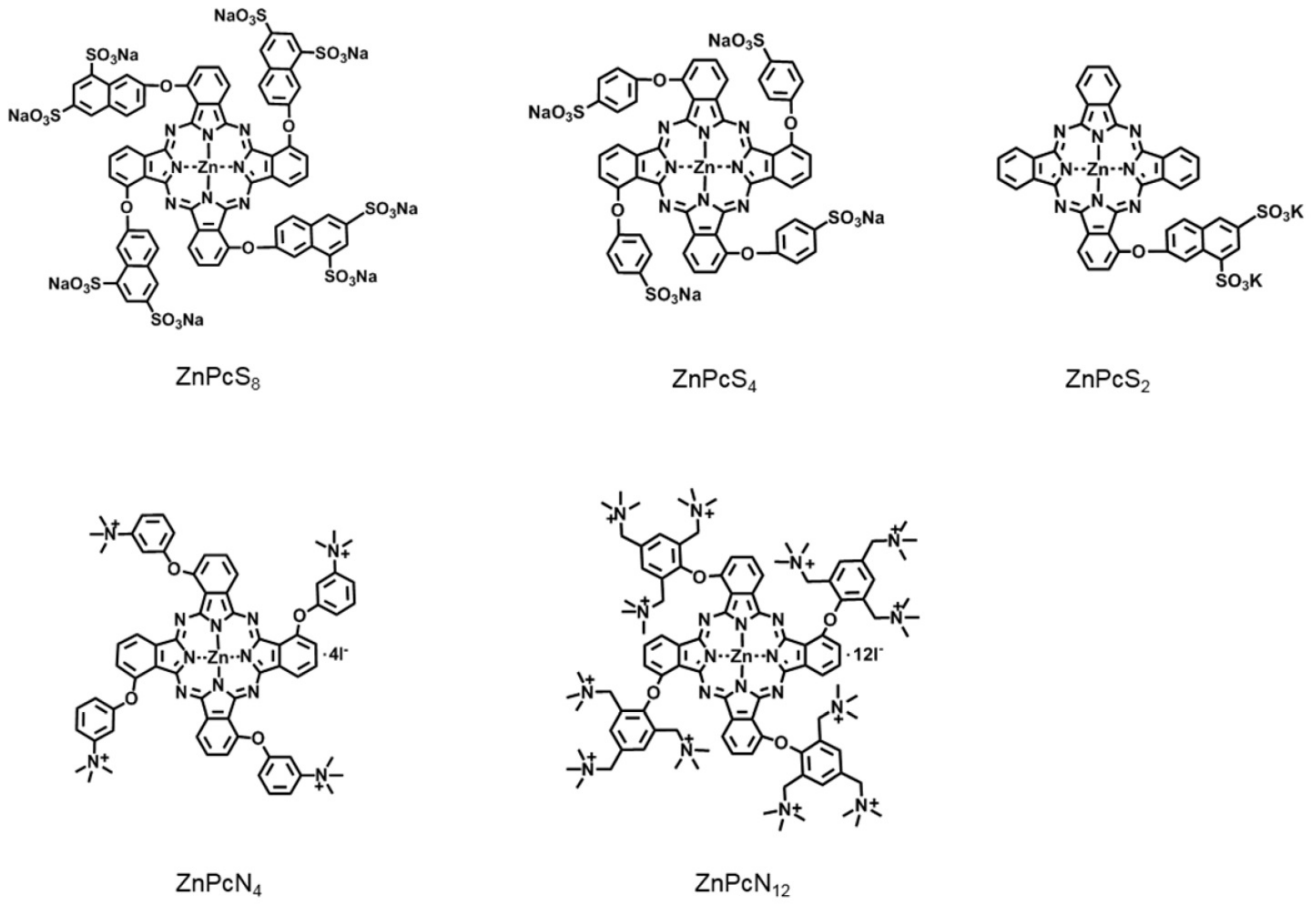
Chemical structures of the phthalocyanines, including ZnPcS_8_, ZnPcS_4_, ZnPcS_2_, ZnPcN_4_, and ZnPcN_12_. Here, only one of the possible C_4h_ isomers is displayed for the tetrasubstituted phthalocyanines.

Zinc(II) phthalocyanines are photosensitizers with high potential for photodynamic therapy (PDT) due to their strong absorption in the far-red/near-infrared regions and high efficiency in generating reactive oxygen species (ROS) 17-21. Recently, several studies reported that zinc(II) phthalocyanines substituted with hydrophilic groups (*e.g.*, carboxyl and sulfonic acids) show strong binding affinity with albumin 22-24_._ Inspired by these reports, we herein attempt to utilize this binding affinity to provide a new approach for targeted PDT. In addition, to systematically study the interaction mechanism between albumin and phthalocyanine molecules, phthalocyanine derivatives bearing different anionic and cationic groups are designed and synthesized (**Figure 1**). Interestingly, we find that all of these water-soluble phthalocyanines can specifically bind to albumin, which in turn enhances their tumor-targeting ability. In particular, the positively charged phthalocyanines selectively bind to albumin dimer over albumin monomer, and the phthalocyanine bearing four cationic groups (ZnPcN_4_) shows outstanding PDT efficacy against tumors in preclinical models.

## Results and Discussion

### Phthalocyanines selectively bind to albumin

**Figure 2:**
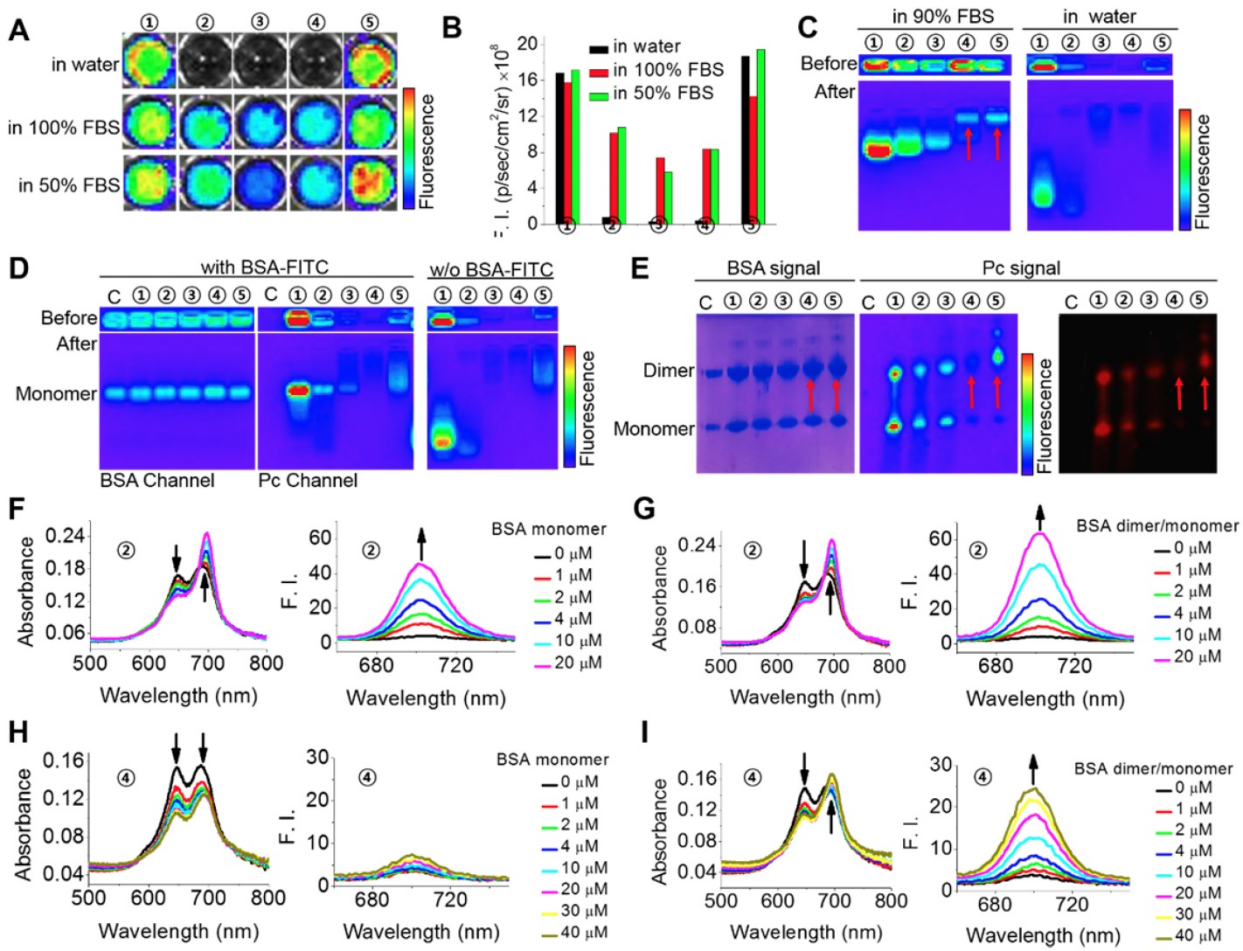
Specific interactions between the phthalocyanines and albumin monomer or dimer. (A-B) Fluorescence images (A) and fluorescence intensities (B) of the phthalocyanines in aqueous solutions containing 0, 50, and 100% FBS. ①: ZnPcS_8_. ②: ZnPcS_4_. ③: ZnPcS_2_. ④: ZnPcN_4_. ⑤: ZnPcN_12_. All the compounds were at 20 μM. The images were obtained when the solutions had stood for 5 min after phthalocyanine dissolution in the media. Fluorescence images were excited at 640 nm and monitored at 720 nm with an IVIS Lumina II imaging system. (C) Gel assays of phthalocyanines in water with and without FBS. All the phthalocyanines were at 2 μM. Gel: 2% agarose gel. Running conditions: 100 V, 20 min. (D) Gel assays of phthalocyanines in PBS with and without BSA monomer (18 μM, labeled with FITC). All the phthalocyanines were at 2 μM. Gel: 2% agarose gel. Running conditions: 100 V, 20 min. FITC was excited at 430 nm and monitored at 520 nm. Phthalocyanines were excited at 640 nm and monitored at 720 nm. C: control, without any phthalocyanine. (E) Native polyacrylamide gel electrophoresis (PAGE) of the phthalocyanines with albumin in PBS (pH 7.4). The gel was stained with Coomassie brilliant blue (CBB) to indicate albumin monomer (~66 kDa) and dimer (~132 kDa) and imaged in both the CBB and phthalocyanine channels. The red arrows indicate the locations of both albumin dimer and ZnPcN_4_ or ZnPN_12_. (F) Absorption and fluorescence spectra of ZnPcS_4_ (2 μM) in water with different concentrations of BSA monomer. (G) Absorption and fluorescence spectra of ZnPcS_4_ (2 μM) in water with different concentrations of BSA dimer/monomer. (H) Absorption and fluorescence spectra of ZnPcN_4_ (2 μM) in water with different concentrations of BSA monomer. (I) Absorption and fluorescence spectra of ZnPcN_4_ (2 μM) in water with different concentrations of BSA dimer/monomer.

To investigate the possible interaction of the phthalocyanines with proteins, we first compared their different fluorescence profiles in pure water and protein-containing solutions. As shown in **Figure 2A-B**, fetal bovine serum (FBS) could obviously enhance the fluorescent intensities of ZnPcS_4_, ZnPcS_2_ and ZnPcN_4_ but caused almost no changes in those of ZnPcS_8_ and ZnPcN_12_. This difference is probably because of their different states in aqueous solution. ZnPcS_8_ and ZnPcN_12_ molecules have enough electrostatic repulsion between them to prevent aggregation-induced quenching. As a result, they already show strong fluorescence emission in pure water. In contrast, ZnPcS_4_, ZnPcS_2_ and ZnPcN_4_ form aggregates in pure water, which may be reduced upon adding FBS. To determine which protein may be involved in these interactions, gel assays and mass analysis were carried out. The results indicated that ZnPcS_8_, ZnPcS_4_ and ZnPcS_2_ most likely bind to the albumin monomer, while ZnPcN_4_ and ZnPcN_12_ most likely bind to the albumin dimer (**Figure 2C** and **Figure S1**). To confirm their binding with the albumin monomer or dimer, pure bovine serum albumin (BSA) monomer and a BSA monomer/dimer (3/7) mixture were utilized for further studies. As shown in **Figure 2D-E**, gel assays confirm that ZnPcS_8_, ZnPcS_4_ and ZnPcS_2_ can bind to both albumin monomer and dimer, while ZnPcN_4_ and ZnPcN_12_ can bind to only albumin dimer. In addition, the results of absorption and fluorescence spectral measurements confirm that both pure BSA monomer and the BSA monomer/dimer mixture can reduce the ZnPcS_4_ aggregates, while only BSA dimer can reduce the ZnPcN_4_ aggregates (**Figure 2F-I**). These results indicate that all five of these phthalocyanines can selectively bind to albumin over other proteins, but only the positively changed compounds (ZnPcN_4_ and ZnPcN_12_) can selectively bind to albumin dimer over albumin monomer. It's also worth to mention that the ROS generation of the phthalocyanines could be increased after binding to albumin (**Figure S2**).

**Figure 3:**
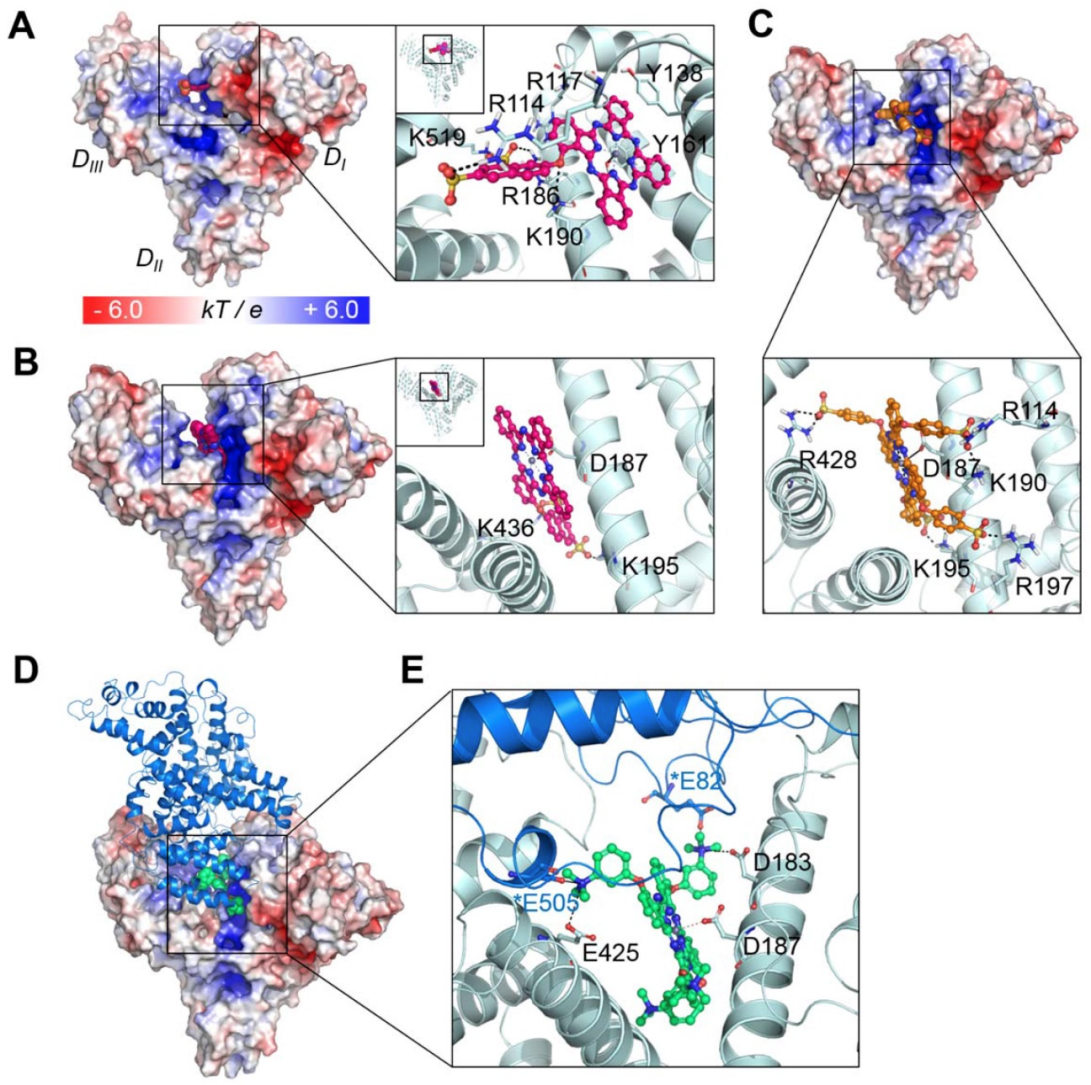
Binding modes of the phthalocyanines with HSA. (A) Binding mode of ZnPcS_2_ at the heme binding site. (B) The other possible binding mode of ZnPcS_2_ at the cleft region of HSA. (C) Binding mode of ZnPcS_4_. The protein surface is colored by the electrostatic potential, and the bound compound is shown as spheres with the carbon atoms in magenta. In the enlarged view, the compound is displayed as a ball-and-stick model, and the interacting residues are displayed as thin sticks with their carbon atoms in light blue. The dative bond between the Zn atom and Y161 or D187 is depicted as a black line, and ionic interactions are marked by black dashed lines. (D) ZnPcN_4_ bound at the HSA dimer interface. The protein surface of one monomer is colored by the electrostatic potential, and the bound compound is shown as spheres with the carbon atoms in lime green. The other monomer is displayed as the marine-colored ribbon. (E) The bound ZnPcN_4_ (lime green, ball-and-stick) is coordinated to D187 via its Zn atom, and the positively charged amine substituents can form ionic interactions with several negatively charged residues (thin sticks, light blue for one monomer and marine for the dimer partner). The interacting residues in the dimer partner are marked by asterisks.

To further investigate the binding mechanism of the compounds, we carried out docking studies, in which their binding conformations to the whole structure of serum albumin were rigorously searched (see Experimental procedures and supporting **Figure S3-4**). We utilized the X-ray crystal structure of human serum albumin (HSA) bound to a heme molecule, which has a similar structural scaffold to that of our compounds (note that for BSA, the appropriate structure for the docking of comparatively large molecules such as ours is not available). Since BSA and HSA show strong similarities in their structures (sequence identity of ~75%, RSMD of ~1 Å) 25, the binding modes in both proteins might be similar. Our results showed that the compound ZnPcS_2_ displayed two different possible binding modes: one bound at the heme binding site with the lowest energy score (**Figure 3A**) and the other bound at the cleft region (**Figure 3B**). At the heme site, the flat phthalocyanine-zinc ring nicely occupies the binding pocket, similar to the scaffold of the heme molecule, and the Zn atom coordinates with the oxygen atom of the Y161 residue. The phthalocyanine-zinc ring also forms a π-stacking interaction with Y138. The linker oxygen atom forms an ionic interaction with K190, and the two sulfonate groups interact with the K519, R114, and R186 residues. The second possible binding mode is the most highly populated conformer and is found in the cleft region, forming a dative bond with D187. The flat phthalocyanine-zinc ring penetrates the cleft site, and the two sulfonate groups establish ionic interactions with K195 and K436.

In the case of ZnPcS_4_, the four bulky substituents make it very difficult for the compound to bind at the heme site. In particular, the heme site located in the D_I_ domain has two disulfide bridges maintaining the structural stability of the helices. Indeed, the structural alignment of the heme site of various albumin structures showed that their architecture is very similar in all structures, suggesting that this region is not highly flexible upon binding various drugs or lipids. Although we consider protein flexibility, it seems to be nearly impossible for the tetrasubstituted phthalocyanine compounds to bind at the heme site. Rather, they showed the lowest energy and most highly populated conformations in the cleft region (**Figure 3C**). Similar to the second binding mode of ZnPcS_2_, this binding mode of ZnPcS_4_ involves the flat phthalocyanine-zinc ring penetrating the cleft site and the Zn atom coordinating with D187. The sulfonate groups of the tetrasubstituted compound form ionic interactions with the surrounding positively charged residues, such as R114, K190, K195, R197, and R428. The distance between the compound's sulfonate oxygen atom and the side chain of W214 (which is the only tryptophan residue of HSA) is ~1.2 nm in the cleft binding complex and ~ 2.6 nm in the heme site binding complex (**Figure S5**). Considering that the typical range of the distance between the acceptor and the donor in fluorescence resonance energy transfer (FRET) is 1 ~ 10 nm 26, both predicted binding modes seem to be reasonable for the fluorescence quenching of the tryptophan in albumin induced by the addition of these phthalocyanines through FRET (**Figure S6**).

The scaffold of ZnPcN_4_ can be similarly fitted to the cleft region, maintaining coordination with D187; however, its positively charged substituents seem to be electrostatically repulsed by the cleft surface (**Figure S7**; note that the electrostatic potential of the cleft region is quite positively charged and that the negatively charged region in the cleft is around the D187 residue, which coordinates with the Zn atom of our compound). This behavior may explain why, in contrast to the negatively charged compounds, which show good electrostatic complementarity to the cleft region, the positively charged compounds cannot bind well to the albumin monomer.

As shown in **Figure 2E**, ZnPcN_4_ and ZnPcN_12_ prefer to bind to the dimer structure, while the negatively charged compounds can bind to both monomer and dimer. It is well known that albumin can form a dimer. In the natural state, albumin exists in equilibrium between the monomer and dimer/oligomer 27. Several studies have reported that increased concentrations of albumin dimer in circulating blood are associated with diseases such as chronic renal disease and oxidative damage in the blood 28-31. However, their association mechanism has not yet been clearly identified. Among the available structural information, we selected the X-ray crystal structure of HSA dimer with the relevant interface contact (PDB id: 3JQZ).^32^ This structure is complexed with lidocaine, a positively charged molecule as our compounds. Interestingly, this cationic drug molecule also occupies the cleft of HSA, and the positively charged amine group interacts with D187, which is shown in our model as the residue coordinating with the zinc atom. Based on this crystal structure, a model of the HSA dimer bound to ZnPcN_4_ was constructed and refined using energy minimization and Monte Carlo (MC) sampling (see Experimental procedures). As shown in **Figure 3D-E**, in addition to the interactions with the monomer, the positively charged substituents of ZnPcN_4_ also form ionic interactions with two glutamate residues (E82 and E505) in the dimer partner. Moreover, the contact interface of the dimer partner is slightly negatively charged, so positively charged compounds can be energetically stable when they bind to the dimer structure.

Collectively, these five phthalocyanines can bind to the HSA cleft region via coordination of their Zn atoms with D187, but the binding mechanisms of negatively and positively charged compounds are quite different. ZnPcS_4_ binds to the monomer with its negatively charged substituents fitting very well to the cleft surface. ZnPcS_4_ can also bind to the dimer, but direct interaction with the dimer partner seems to be negligible. While ZnPcN_4_ can be fitted to the monomer cleft region, the positively charged substituents can be electrostatically repulsed by the surface. In the dimer structure, ZnPcN_4_ can form ionic interactions with the dimer partner, which may contribute to the more stable and preferable binding of this compound to the dimer.

### *In vivo* albumin as a natural carrier that enhances the tumor-targeted delivery of phthalocyanines

**Figure 4:**
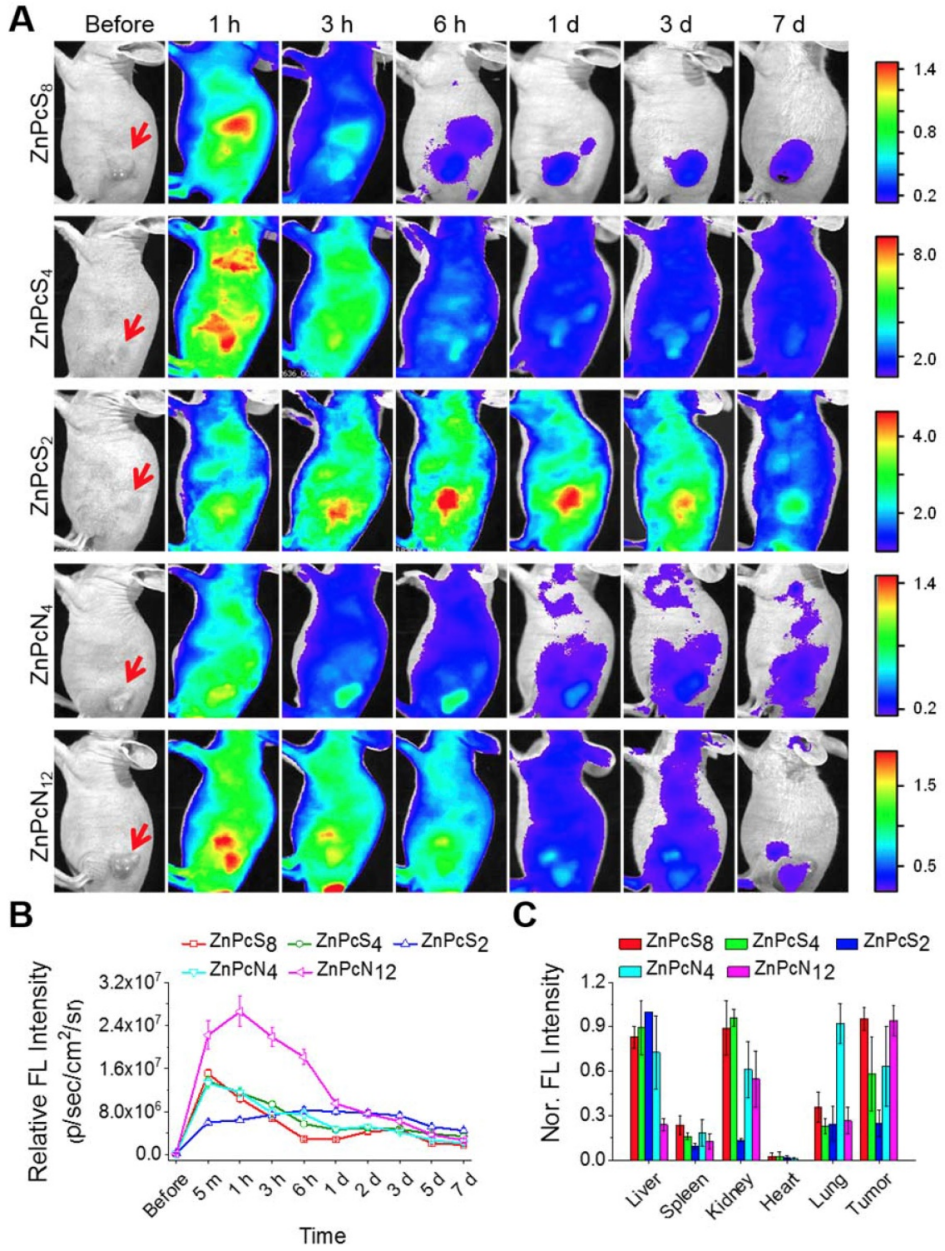
Biodistribution of the phthalocyanines in SCC-7 tumor-bearing mice. (A) *In vivo* fluorescence images of SCC7 tumor-bearing mice upon tail vein injection of the aqueous phthalocyanine solutions (200 μL, 200 μM). The red arrows indicate the tumor location. (B) Temporal tumor accumulation profiles of the phthalocyanines, as determined by fluorescence intensities from the tumor in *in vivo* images. (C) Biodistribution of the phthalocyanines, as determined *ex vivo* by fluorescence intensities from the tumor and other organs excised at 7 d postinjection.

As introduced above, *in vitro*-processed albumin has been widely used to construct carrier systems for drug delivery. Since our phthalocyanines show strong binding affinities for albumin, we thus hypothesized that upon *in vivo* administration, they bind to endogenous albumin molecules spontaneously to form an *in situ* delivery system where albumin serves as an intrinsic biomolecular carrier. To prove this concept, the phthalocyanine compounds were administered into SCC7 (squamous cell carcinoma) tumor-bearing mice via intravenous injection, and their biodistribution was monitored temporally by *in vivo* fluorescence imaging. As revealed in **Figure 4A-B** and** Figure S8**, all the compounds were shown to spread throughout the body immediately after injection and fade gradually with time, indicative of their efficient circulation in the blood stream. Concomitantly, they accumulated rapidly and stayed longer at the tumor site, allowing clear visualization of the tumor location *in vivo*. The biodistribution results determined by *ex vivo* imaging revealed that a significant amount of the phthalocyanine compounds were retained at the tumor site even at 7 d postinjection in spite of their rapid excretion from other parts of the body (**Figure 4C**). The consequent tumor-targeting ability is most likely attributable to the targeted transport by *in vivo* albumins via spontaneous binding of the phthalocyanines, where passive targeting would make an additional contribution in the case of dimer-binding ZnPcN_4_ and ZnPcN_12_ owing to the larger intrinsic carrier (albumin dimer, ~130 kDa), which can promote the size-dependent EPR effect. Efficient tumor accumulation of the phthalocyanines was also observed in a different type of tumor xenograft mouse model (HT-29, human colorectal adenocarcinoma), suggesting that the endogenous albumin-mediated delivery system can be a general strategy for systemic tumor targeting (**Figure S9**). Notably, methylene blue, a clinically used blue dye with negligible albumin-binding ability, did not show such tumor-targeting behavior upon systemic injection by itself (**Figure S10**), further corroborating that the spontaneous *in vivo* binding with albumin plays a crucial role in the tumor-targeting ability of our phthalocyanines.

### PDT efficiency of the phthalocyanines in tumor-bearing mice

**Figure 5:**
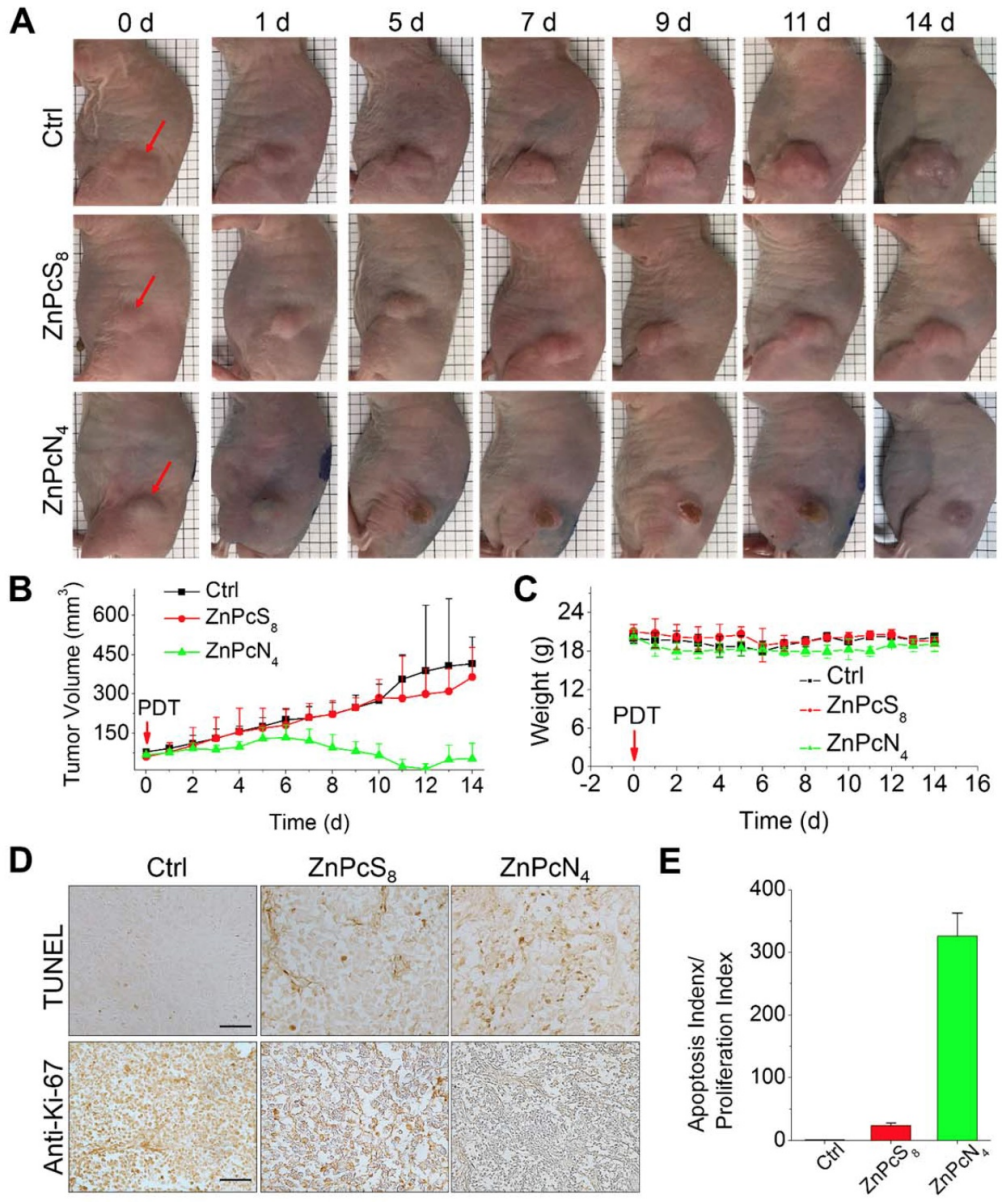
*In vivo* PDT treatment of HT-29 tumor-bearing mice with phthalocyanines. (A) Representative photographs of PDT-treated mice. For PDT treatment, laser irradiation (655 nm, 30 min) of the tumor tissue was performed at 3 h postinjection of the phthalocyanines (200 μL, 200 μM). In the control group, mice were exposed to laser irradiation after PBS (200 μL, pH 7.4) injection. The red arrows indicate the tumor location. (b-c) Temporal changes in tumor volume (B) and mouse body weight (C) during PDT treatments. (D) Histological images of tumor tissues resected from the mice 1 d after PDT treatment. Tumor sections were prepared for anti-Ki-67 immunohistochemical and TUNEL analyses. (E) Quantitative analysis of apoptosis and proliferation in tumor tissues. Apoptosis and proliferation indices were determined by the number of TUNEL- and Ki-67-positive cells in the histological images, respectively.

Encouraged by the *in vivo* tumor-targeting results, we next evaluated the phototherapeutic efficacy of these phthalocyanines by monitoring tumor growth inhibition after PDT treatment. To examine the effect of differently charged substituents, which were previously shown to determine the major binding partner (albumin monomer or dimer), we chose monomer-binding ZnPcS_8_ and dimer-binding ZnPcN_4_ for comparison and performed a single operation of PDT at 3 h postinjection, when the two compounds displayed similar levels of tumor accumulation (**Figure 4B**). In the operation, the phthalocyanines were intravenously injected into HT-29 tumor-bearing mice (tumor volume: 60-80 mm^3^), and the tumors were exposed to red laser irradiation (655 nm, 200 mW, 30 min) at 3 h postinjection. As shown in **Figure 5A**, the mouse group treated with dimer-binding ZnPcN_4_ presented apparent signs of tumor necrosis after PDT treatment, where the tumor surface manifested redness followed by scab formation within 5 d after PDT treatment. During the 2 week monitoring period, the tumor growth in that group was significantly suppressed, indicating successful PDT treatment with dimer-binding ZnPcN_4_ (**Figure 5B**). In addition to its therapeutic efficacy, ZnPcN_4_ caused no noticeable body weight loss in the treated mice, implying minimal toxic impacts on animal viability (**Figure 5C**). Interestingly, monomer-binding ZnPcS_8_ did not show such a therapeutic effect even though its tumor accumulation level was observed to be similar to that of dimer-binding ZnPcN_4_ in the *in vivo* and *ex vivo* images (**Figures 4B** and **S11**). In sharp contrast to the tumor inhibitory effect of ZnPcN_4_, the group treated with ZnPcS_8_ showed rapid tumor growth at a similar rate to that of the control group treated with PBS (pH 7.4), with no sign of tissue response to PDT (**Figure 5A-B**).

**Figure 6:**
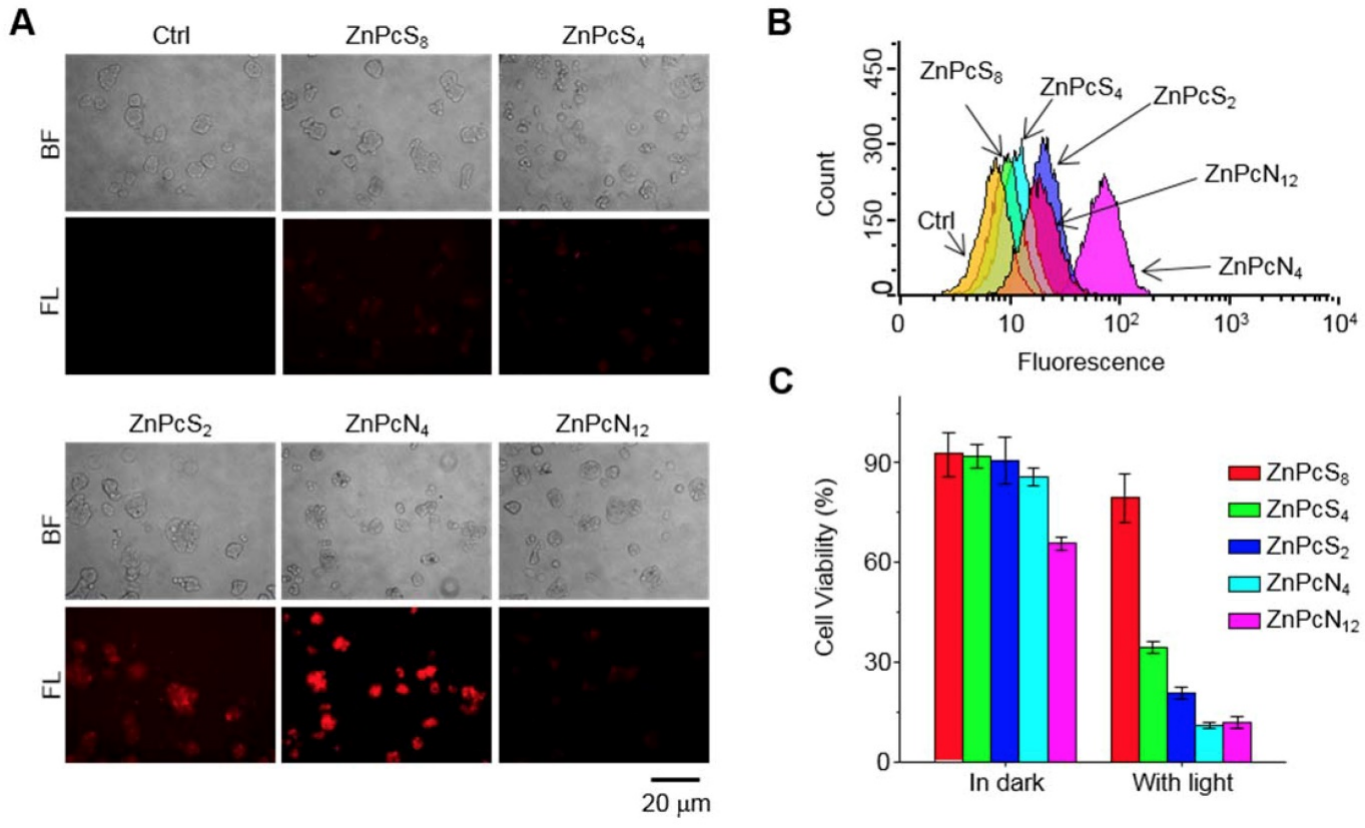
Intracellular delivery efficiency and photoinduced cytotoxicity of the phthalocyanines. (A-B) Fluorescence microscopic images (A) and flow cytometry analysis (B) indicating the uptake of phthalocyanines (20 μM) by HT-29 cells following 1 h of incubation. The images were obtained by using a filter set (excitation: 620 ± 30 nm, emission: 690 ± 30 nm). (C) Photoinduced cytotoxicity of phthalocyanines against HT-29 cells evaluated by the colorimetric MTT assay. Cells were exposed to laser irradiation (655 nm, 10 min) after intracellular delivery of the phthalocyanines.

The distinct therapeutic responses observed above could be further confirmed by histological analysis of the tissue sections prepared from the tumors excised 1 d after each PDT treatment (**Figure 5D-E**). The tumor sections were stained for terminal deoxynucleotidyl transferase-mediated nick end labeling (TUNEL) and anti-Ki-67 immunohistochemical analyses, which allowed us to quantify the degree of apoptosis normalized by the degree of proliferation. It was observed that the tumor sections from the ZnPcN_4_-treated group presented a 14-fold higher ratio of apoptosis index (TUNEL-positive cells)/proliferation index (Ki-67-positive cells) than those from the ZnPcS_8_-treated group, evidencing the remarkably efficient phototherapy enabled by dimer-binding ZnPcN_4_. These distinct PDT responses may be attributable to the different cell uptake efficiencies of phthalocyanines; i.e., in the presence of albumin-containing serum (FBS), highly negative ZnPcS_8_ exhibited the lowest cell uptake level and in turn the lowest PDT effect, whereas moderately positively charged ZnPcN_4_ is superior in both cell uptake and phototoxicity while maintaining cell viability in the absence of light (**Figure 6**). Note that the PDT treatment with highly positive ZnPcN_12_ caused severe pathological changes in the visceral organ tissues and that the mouse survival rate was decreased to zero at 3 d after PDT treatment, indicating acute toxicity (**Figure S12**). The *in vivo* toxicity of ZnPcN_12_ is attributable to the highly positively charged molecular structure 33,34 and the consequently strong dyeing effect on normal tissues around the tumor (**Figure 4**), which could cause collateral damage and toxicities when light diffuses through the tissue upon laser irradiation of the tumor. All these results suggest that among others, moderately positive dimer-binding ZnPcN_4_ holds promise as an *in situ* albumin-binding photosensitizer for efficacious PDT of cancer by external carrier-free tumor targeting.

## Conclusion

In summary, we have developed five water-soluble phthalocyanines that show strong binding affinity for albumin. Interestingly, we found for the first time that the two positively charged compounds could selectively bind to albumin dimer over albumin monomer, while the three negatively charged phthalocyanines could bind to both albumin monomer and dimer. The binding modes of the phthalocyanines to HSA were well simulated, which should inspire increasing interest in the design and development of albumin monomer-targeted or albumin dimer-targeted probes or drug delivery strategies. Based on our interesting findings, we further demonstrated a green and efficient delivery approach using the native albumin *in vivo* as the carrier to enhance the tumor-targeting ability of these phthalocyanines. In particular, the phthalocyanine bearing four cationic groups, ZnPcN_4_, displays outstanding PDT efficacy against tumors.

## Materials and Methods

**Chemical materials and instruments.** Methylene blue (MB), phthalonitrile, 1,8-diazabicyclo-[5.4.0]undec-7-ene (DBU), zinc acetate and BSA monomer were purchased from Sigma-Aldrich. ZnPcS_8_, ZnPcS_4_ and ZnPcN_12_ were prepared using our previously described procedure 35-37. The synthetic details of ZnPcS_2_ and ZnPcN_4_ are shown in the supplementary materials.

To obtain the BSA dimer/monomer (7/3) mixture, BSA (3.7 g, Sigma-Aldrich) in distilled water (70 mL) was incubated with Darco activated charcoal (3.5 g) washed with distilled water 38. The BSA-charcoal mixture was titrated with 0.2 N HCl below pH 3.0 on ice. After incubation for 1 h, the supernatant was separated from the lipid-attached charcoal by centrifugation and was subsequently neutralized to pH 7.0 by adding 0.2 N NaOH. The lipid-free proteins were concentrated and then loaded onto a HiLoad 16/600 Superdex-75pg (GE Healthcare, USA) pre-equilibrated with A-buffer (50 mM Tris, 150 mM NaCl, pH 8.0). The fractions containing the BSA dimer/monomer mixture were collected and diluted 10-fold with B-buffer (50 mM Tris, pH 8.0). The diluted fraction was loaded onto a HiTrap Q Fast Flow (GE Healthcare, USA) pre-equilibrated with B-buffer. The BSA dimer/monomer mixture was eluted between 380 mM and 500 mM NaCl and concentrated for loading onto a HiLoad 16/600 Superdex-200pg (GE Healthcare, USA) pre-equilibrated with A-buffer. The final BSA dimer/monomer fraction was concentrated to 27 mg/mL along with desalting against PBS buffer. The BSA dimer/monomer mixture was stored at -80 °C. During all purification procedures, the purity and homogeneity of the purified protein were determined based on native-PAGE analysis.

Electronic absorption spectra were recorded on a Shimadzu UV-2450 spectrophotometer. Fluorescence spectra were obtained on an Edinburgh FL900/FS900 spectrofluorometer.

**Gel assays.** The albumin-binding affinity of phthalocyanines was evaluated by gel retardation assay. For the experiments, a mixture of phthalocyanines (2 μM) and albumin (18 μM, a mixture of albumin monomer and albumin dimer) in PBS containing 10% DMSO was loaded onto a 10% native polyacrylamide (PAGE) gel, and electrophoresis was performed in Tris-glycine buffer solution at 110 V. After electrophoresis, CBB-stained albumin and phthalocyanine bands were visualized using a gel documentation system (Slimlight 5000K-M, Korea) and an IVIS Spectrum imaging system (PerkinElmer, USA), respectively. Furthermore, the mixture of phthalocyanines (2 μM) and FITC-labeled BSA monomer (18 μM) in PBS (pH 7.4) containing 10% DMSO was loaded onto 2% agarose gel, and electrophoresis was performed in TAE buffer solution at 100 V. After electrophoresis, the gel was imaged using an IVIS spectrum imaging system.

**Fluorescence titration.** The binding constant of phthalocyanines with albumin was measured by fluorescence titration (Figure S6). The fluorescence spectra (excited at 280 nm) of albumin (3 μM) in water after adding different concentrations of phthlocyanines were measured. Then, their fluorescence intensity at 344 nm were recorded and used to calculated the binding constant according to the double logarithm regression curve, Log((F_0_-F)/F)=LogK+nLog[Pc] 39.

**Computational studies.** Protein and ligand preparations. The X-ray crystal structures of HSA complexed with heme molecule (PDB codes: 1N5U 40, 1O9X 41 ) were prepared using the Protein Preparation Wizard in Maestro, version 9.2 (Schrödinger, LLC, NY, USA). During the preparation process, bond orders were assigned, hydrogen atoms were added, and protonation states of the residues at pH 7.4 were generated by Epik, version 2.6. All the hydrogen atoms were energy minimized with the optimized potential for liquid simulation (OPLS) 2005 force field until the average root-mean-square deviation (RMSD) for hydrogen atoms reached 0.30 Å. Since the metal-coordinated ligand molecule cannot be appropriately prepared with the conventional ligand preparation tool, metal coordination in the phthalocyanine molecule was prepared using the Protein Preparation Wizard. The resulting structures were energy minimized using the implicit solvent and OPLS 2005 force fields.

Blind docking and binding mode selection. The ligands were docked into several binding pockets of HSA with Glide, version 6.1, in Maestro using the following steps: (i) Initially, the grid box was generated using the centroid of the cocrystallized ligand. We set the grid box size large enough to cover all the binding cavities in HSA. (ii) For the docking stage, Glide SP (standard precision) docking, which searches for up to 500 conformations per ligand, was performed. (iii) The docked conformations were clustered based on their RMSDs, and the final modes were selected based on their scores and populations.

Dimer modeling. The positively charged compounds were docked to the HSA monomer first, and then the dimer model was generated using the available HSA homodimer crystal structure complexed with lidocaine (PDB code: 3JQZ) 32. Since this crystal structure's binding site is too small relative to our compound, the original docking to the PDB structure (3JQZ) failed to retrieve the solution. The complex structure of ZnPcN_4_ and HSA monomer was aligned to the dimer crystal structure, and then the monomer model and the ligand-free dimer partner (chain B) of 3JQZ.pdb were merged. The merged chain B was further refined using energy minimization and MC sampling. The OPLS3 force field 42 and the variable-dielectric generalized Born (VSGB) solvation model were used during the simulation. After 10000 steps of MC sampling, the lowest energy model structure was obtained.

All computational studies were undertaken on an Intel Xeon octa-Core 2.5 GHz workstation with Linux CentOS, release 5.8, and the molecular graphics figures were generated by PyMOL software (http://www.pymol.org).

**Intracellular delivery into cancer cells.** HT-29 cells were seeded onto 35-mm cover glass bottom dishes (1ⅹ10^5^ cells per dish). Once they reached a confluence of 70-80%, cells were washed twice with PBS (pH 7.4) and treated with phthalocyanines (20 μM in RPMI 1640 medium). After 1 h of incubation, the cells were washed twice with PBS (pH 7.4) and recharged with fresh media. Cells were imaged by using a LEICA DMI3000B fluorescence microscope equipped with a Nuance FX multispectral imaging system (CRI, USA).

To quantitatively evaluate the intracellular delivery efficiency, HT-29 cells were seeded on 6-well plates (1×10^6^ cells per well). After 24 h of incubation, the cells were treated with phthalocyanines (20 μM in RPMI 1640 medium) for 1 h. Then, the cells were washed twice with PBS (pH 7.4), trypsinized, and collected by centrifugation. The collected cells were analyzed by a Guava easyCyteTM flow cytometer (EMD Millipore, USA).

**Photo-induced cytotoxicity against cancer cells.** HT-29 cells were seeded in 96-well plates (1ⅹ10^4^ cells per well). Once they reached a confluence of 70-80%, the cells were treated with phthalocyanines (20 μM in RPMI1640 medium). After 1 h of incubation, the cells were recharged with fresh media, exposed to laser irradiation (655 nm, 200 mW, 10 min), and further incubated for 24 h. Cell viability was evaluated via the colorimetric MTT assay 43.

***In vivo* and *ex vivo* imaging of tumor accumulation.** The animal study was performed according to the guidelines offered by the Korea Institute of Science and Technology (KIST). For animal experiments, BALB/c nude mice (5-week-old male; Orient Bio Inc., Korea) were anesthetized with intraperitoneal injection of a mixture of Zoletil-Rompun. Tumors were established by subcutaneous inoculation of SCC7 (1 × 10^6^ cells in 60 μL of culture medium) or HT-29 (1 × 10^7^ cells in 100 μL of culture medium) cells into mice. All *in vivo* fluorescence images were taken at designated time points for 1 week after intravenous injection of the aqueous phthalocyanine solution (200 μL, 200 μM) with an IVIS Spectrum imaging system. For *ex vivo* fluorescence imaging, organs including tumors were excised at 7 d postinjection of the phthalocyanines from mice and imaged with an IVIS Spectrum imaging system.

***In vivo* photodynamic therapy.** The aqueous phthalocyanine solution (200 μL, 200 μM) was intravenously injected into HT-29 tumor xenograft mice (n = 4 per group). At 3 h postinjection, tumors were exposed to laser irradiation at 655 nm for 30 min. The tumor volumes were measured and calculated with a × b^2^/2, where a and b are the largest and smallest diameters of the tumor, respectively. For histological examination, tumors were excised from the mice 1 d after PDT treatment with phthalocyanines to be fixed in neutral buffered formalin and embedded in paraffin. The tissue blocks were cut into 10 μm sections and stained for anti-Ki-67 immunohistochemical and terminal deoxynucleotidyl transferase-mediated nick end labeling (TUNEL) analyses. Proliferation and apoptosis indices were determined by quantitation of Ki-67- and TUNEL-positive cells, respectively 44.

**Evaluation of biocompatibility.** For the *in vivo* toxicity study, the visceral organs were excised 1 d after PDT treatment with phthalocyanines. The tissue blocks of the organs were prepared according to the same procedure as the one explained above for tumor block preparation, and hematoxylin (H) and eosin (E) staining was performed to assess the extent of the pathological changes. Photomicrographs of tissue sections were acquired using an Olympus BX51 microscope and image transfer software (Olympus, Tokyo, Japan).

## Supplementary Material

Supplementary figures and the synthesis procedures of the compounds.
