# Supplementary material for: Water-Soluble Phthalocyanines Selectively Bind to Albumin Dimers: A Green Approach Toward Enhancing Tumor-Targeted Photodynamic Therapy
Source: Theranostics. 2019 Aug 14;9(22):6412–23. doi: 10.7150/thno.35210 (PMC6771247; doi:10.7150/thno.35210)
Supplement: Supplementary file 1 — Supplementary figures and the synthesis procedures of the compounds. [file thnov09p6412s1.pdf]

Supporting Information

Water-Soluble Phthalocyanines Selectively Bind to Albumin Dimers: A Green Approach Toward Enhancing Tumor-Targeted Photodynamic Therapy

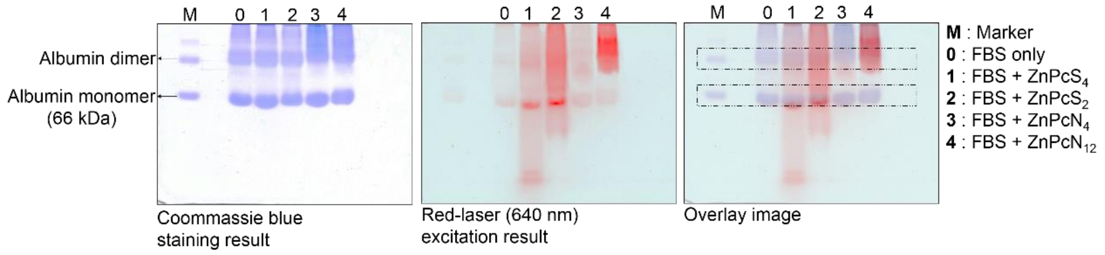

Figure S1. Gel assays indicate that the phthalocyanines can specifically bind to serum albumin. Gel: 10% acryl/bis-acrylamide native gel. Running condition: 100 V, 2 h. All the compounds were at 200 μM.

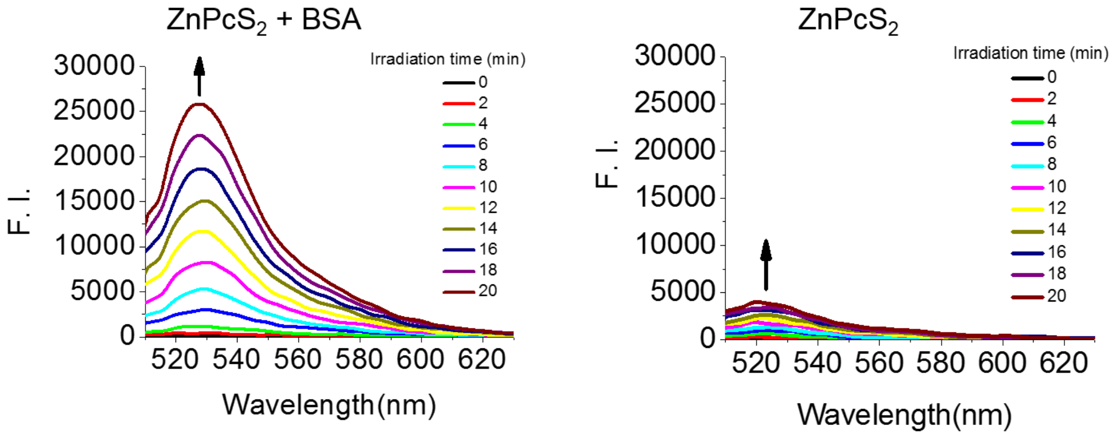

Figure S2. The ROS generation of $ZnPcS_2$ in water with and without albumin detected by using 2,7-dichlorofluorescin diacetate as a probe. The fluorescence of ROS probe was excited at 504 nm.

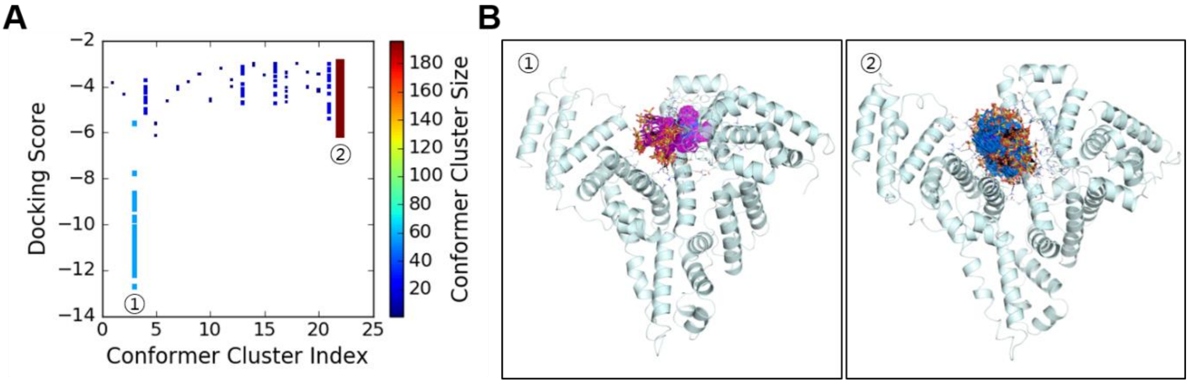

Figure S3. Blind docking result of $ZnPcS_2$. (A) Docking score distribution of the conformer clusters. The 500 generated binding conformers of $ZnPcS_2$ were clustered by their RMSDs. The spot color and size are based on the cluster size. The lowest binding score conformer and the most highly populated conformer are marked as ① and ②, respectively. (B) The lowest binding score conformers and the most highly populated conformers are displayed as sticks with their carbon atoms in purple and sky blue, respectively.

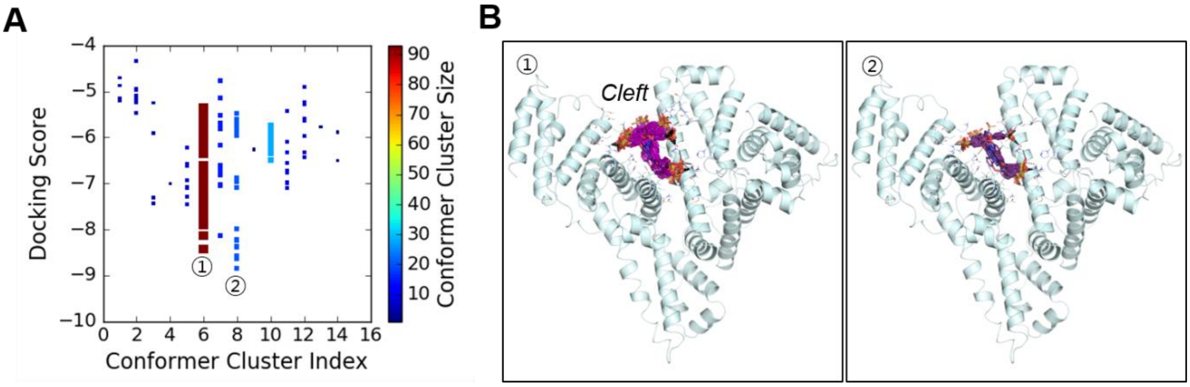

Figure S4. Blind docking result of $ZnPcS_4$. (A) Docking score distribution of the conformer clusters. The 500 generated binding conformers of $ZnPcS_4$ were clustered by their RMSDs. The spot color and size are based on the cluster size. The most highly populated cluster and lowest scored cluster are marked as ① and ②, respectively. (B) The lowest binding score conformers and the most highly populated conformers are displayed as sticks with their carbon atoms in purple and dark purple, respectively. Both clusters are the conformations bound to the cleft region.

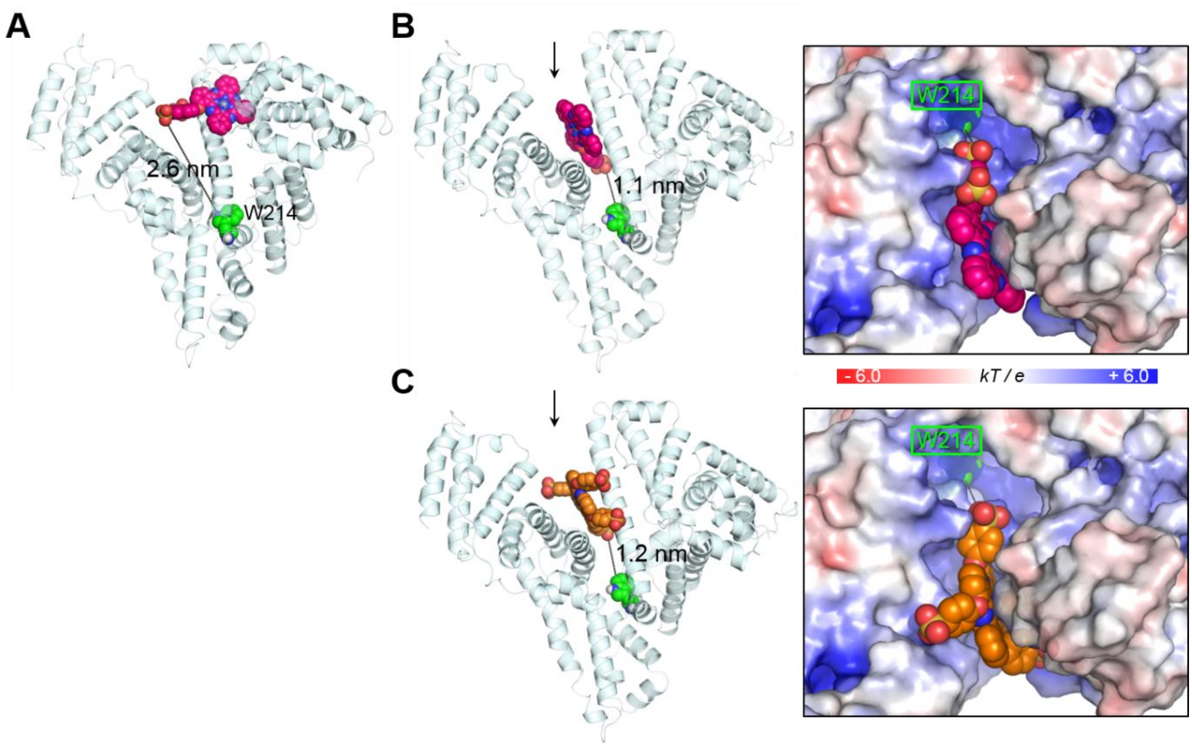

Figure S5. Distances from the tryptophan residue in (A) the binding mode of $ZnPcS_2$ (magenta) at the heme binding site, (B) the binding mode of $ZnPcS_2$ (magenta) at the cleft region, and (C) the binding mode of $ZnPcS_4$ (orange). The W214 residue is shown as spheres with carbon atoms in green. In panels (B) and (C), the top view is shown with the protein surface whose cavities are well occupied by the bound ligands. The protein surface is colored by the electrostatic potential.

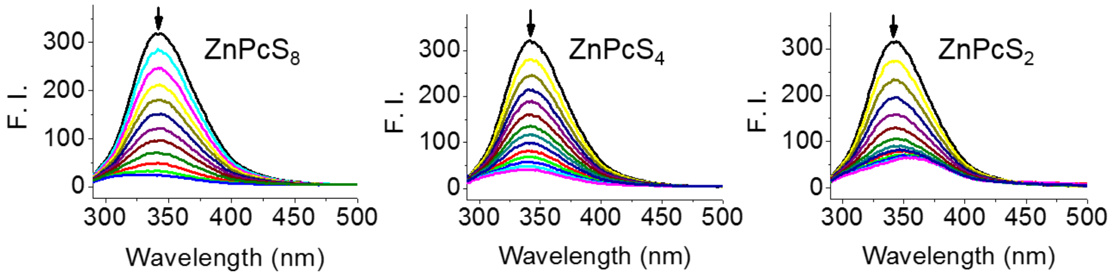

Figure S6. Fluorescence spectra (excited at 280 nm) of BSA (3 μM) after adding different concentrations of $ZnPcS_8$, $ZnPcS_4$, and $ZnPcS_2$.

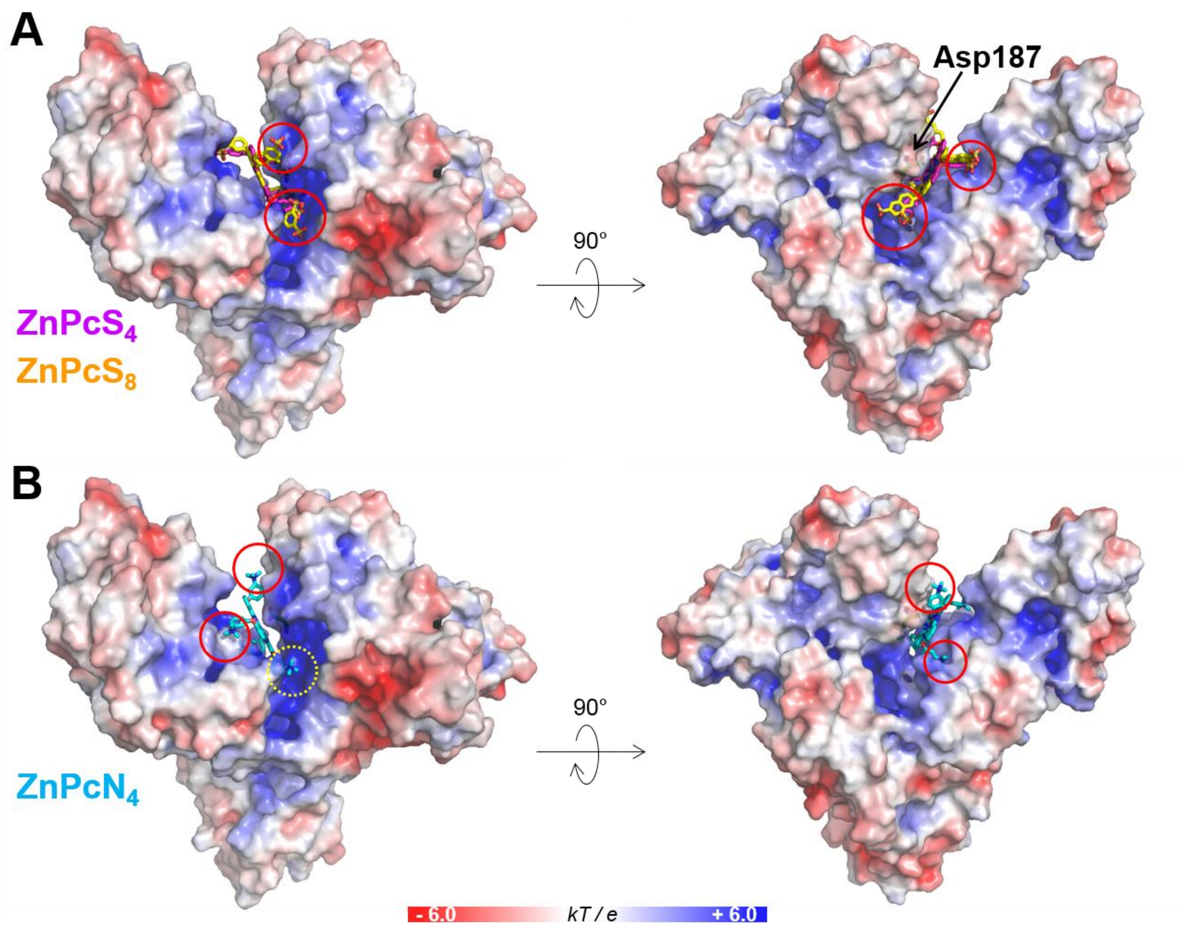

Figure S7. Electrostatic potential of the HSA surface overlaid with the docked conformations of (A) the negatively charged compounds, i.e., $ZnPcS_4$ (purple) and $ZnPcS_8$ (yellow), and (B) the positively charged compound, i.e., $ZnPcN_4$ (cyan). The left panel is the same view as Figure 3, and the right panel is for the opposite side. The location of the D187 residue, which coordinates with the Zn atom, is marked with a black arrow. The negatively charged substituents of the ZnPcS compounds can strongly interact with the cleft surface region (marked by red circles). In the case of ZnPcN4, positively charged substituents can be repulsed by the cleft surface (see the yellow dotted circle).

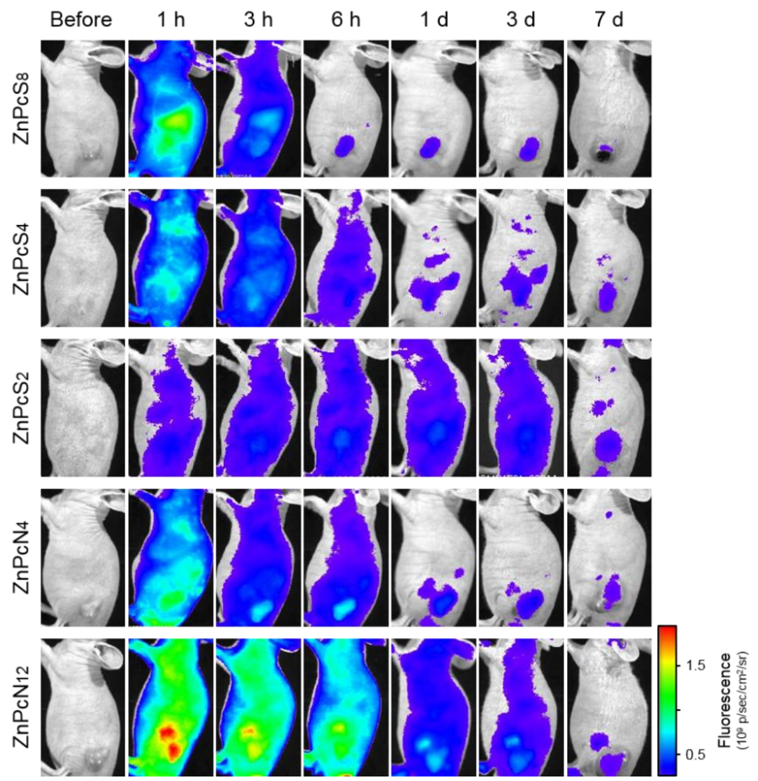

Figure S8. *In vivo* fluorescence images of SCC7 tumor-bearing mice upon tail vein injection of the aqueous phthalocyanine solutions (200 μL, 200 μM). For overall comparison of fluorescence intensities, the supplementary *in vivo* images with all the same scale bar were displayed here.

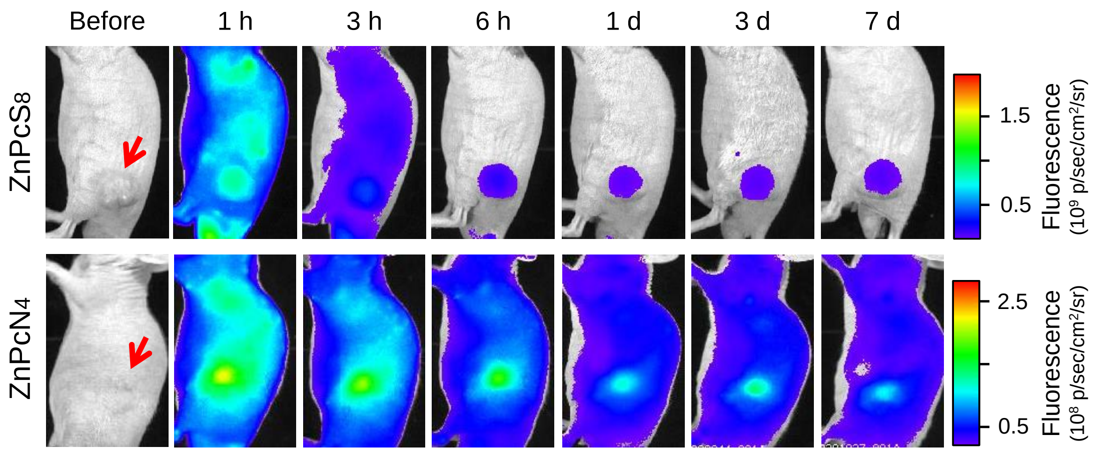

Figure S9. *In vivo* fluorescence imaging of HT-29 tumor-bearing mice upon intravenous administration of aqueous phthalocyanine solutions (200 μL, 200 μM).

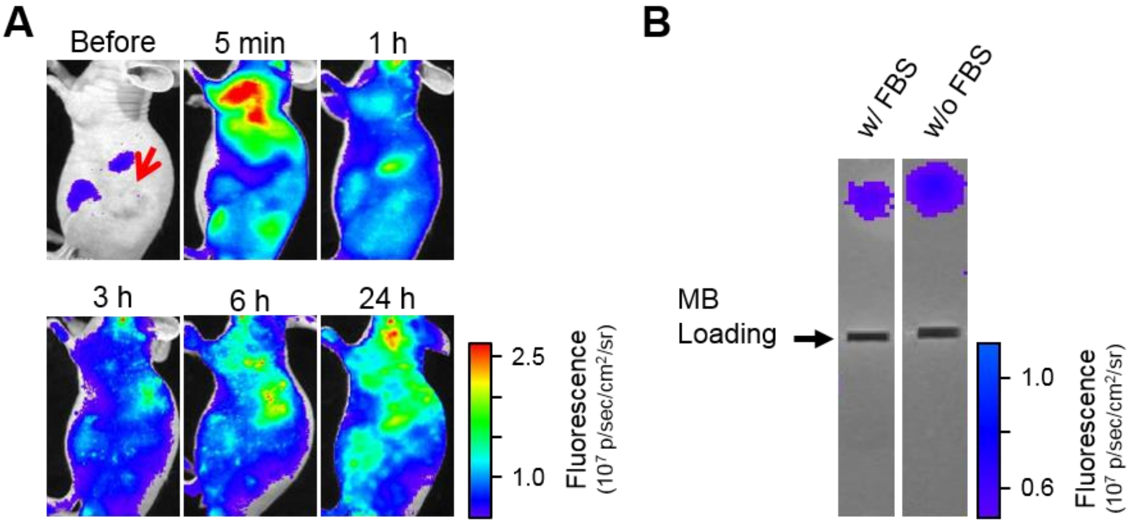

Figure S10. (A) *In vivo* fluorescence imaging of SCC7 tumor-bearing mice upon intravenous administration of an aqueous methylene blue solution (MB, 200 μL, 200 μM). (B) Agarose gel electrophoresis of MB (2 μM).

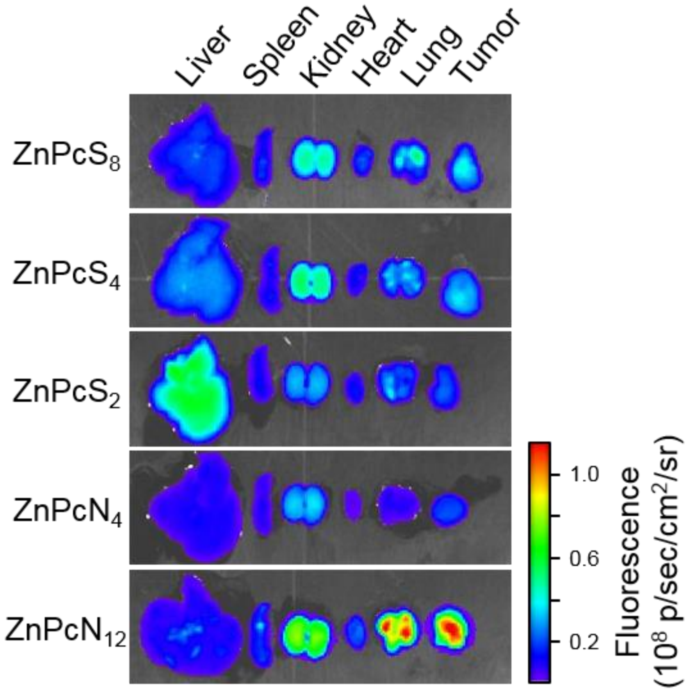

Figure S11. *Ex vivo* fluorescence imaging of the organs excised from the mice bearing tumors at 3 h postinjection of the aqueous dispersion of phthalocyanines.

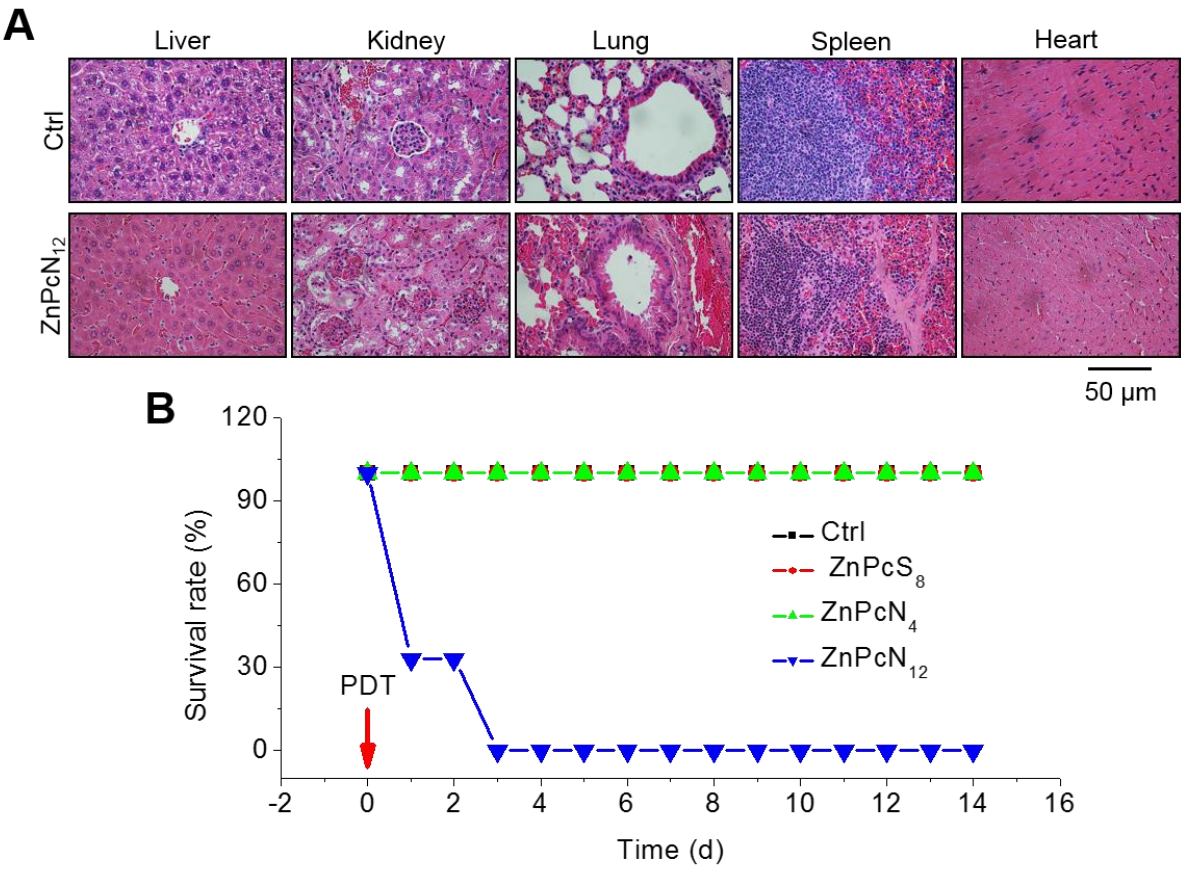

Figure S12. *In vivo* toxicity of the phthalocyanines. (A) Histological images of organs resected from the mice 1 d after PDT treatment with the phthalocyanines. (B) Temporal changes in the mouse survival rate during PDT treatments. In the control group, mice were exposed to laser irradiation after PBS injection (200 μL, pH 7.4).

Synthesis and characterization of ZnPcS$_2$

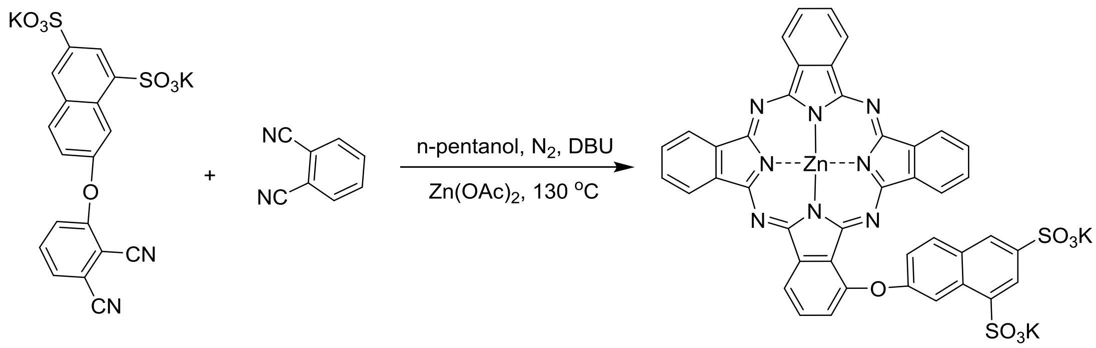

A mixture of 3-[6,8-potassium disulfonate-2-naphthyloxy]phthalonitrile (1.5 mmol) and phthalonitrile (7.5 mmol) in n-pentanol (30 mL) was stirred at 90 °C under an atmosphere of nitrogen for 30 min, and then zinc acetate (4.9 mmol), anhydrous K_2CO_3 (1.5 mmol) and 1,8-diazabicyclo-[5.4.0]undec-7-ene (DBU) (1 mL) were added. The resulting mixture was stirred at 130 °C for 20 h. After cooling, the reaction mixture was filtered. After collecting the filtrate and concentrating, the crude product was first purified by silica gel column chromatography using EA/DMF (10/1, 2/1 and 1/1) as the eluent to generate a green/blue crude product. The

mixture was concentrated under reduced pressure and purified by size exclusion chromatography on a Bio-Beads S-X3 column using DMF as the eluent. The crude product was further purified by recrystallization from a mixture of ethanol and water to afford a bluish green solid $ZnPcS_2$ (98.5 mg, 10.3%). ^{1}H NMR (400 MHz, DMSO-d_6): δ = 9.38-9.47 (m, 4 H, Pc-H_α); 9.32 (d, J = 7.6 Hz, 1 H, Pc-H_α); 9.04 (d, J = 2.4 Hz, 1 H, Pc-H_α); 9.00 (d, J = 7.6 Hz, 1 H, Pc-H_α); 8.25-8.35 (m, 5 H, Pc-H_β); 8.11-8.16 (m, 3 H, Pc-H_β); 8.02-8.08 (m, 2 H, Ar-H); 7.93-7.95 (m, 2 H, Ar-H); 7.68 (dd, J_1 = 2.4 Hz, J_2 = 11.6 Hz, 1 H, Ar-H). HRMS (ESI): m/z Calcd for $C_{42}H_{20}N_8O_7S_2Zn$ $[M-2K]^{2-}$ 438.0088, found 438.0100.

Synthesis and characterization of $ZnPcN_4$

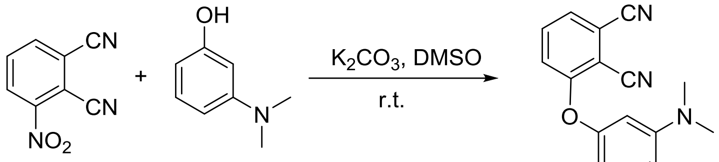

A mixture of 3-nitrophthalonitrile (15.0 mmol), 3-(dimethylamino)phenol (15.0 mmol), and anhydrous K_2CO_3 (30.0 mmol) in DMSO (30 mL) was stirred at room temperature for 72 h under an atmosphere of nitrogen. The reaction mixture was poured into water (150 mL) and standed for 3 h. The brown solid was filtered and washed with water, and then drying in vacuo. The product was afforded as a light brown solid (3.8 g, 96 %). ^{1}H NMR (300 MHz, DMSO-d_6): δ = 7.79-7.81 (m, 2 H, Ar-H), 7.21-7.30 (m, 2 H, Ar-H), 6.67 (dd, J = 2.4 Hz, J = 8.1 Hz, 1 H, Ar-H), 6.55 (t, J = 2.1 Hz, 1 H, Ar-H), 6.44 (dd, J = 1.8 Hz, J = 7.5 Hz, 1 H, Ar-H), 2.91 ppm (s, 6 H, $-CH_3$). HRMS (ESI): m/z Calcd for $C_{16}H_{14}N_3O$ $[M+1H]^+$ 264.1131, found 264.1150.

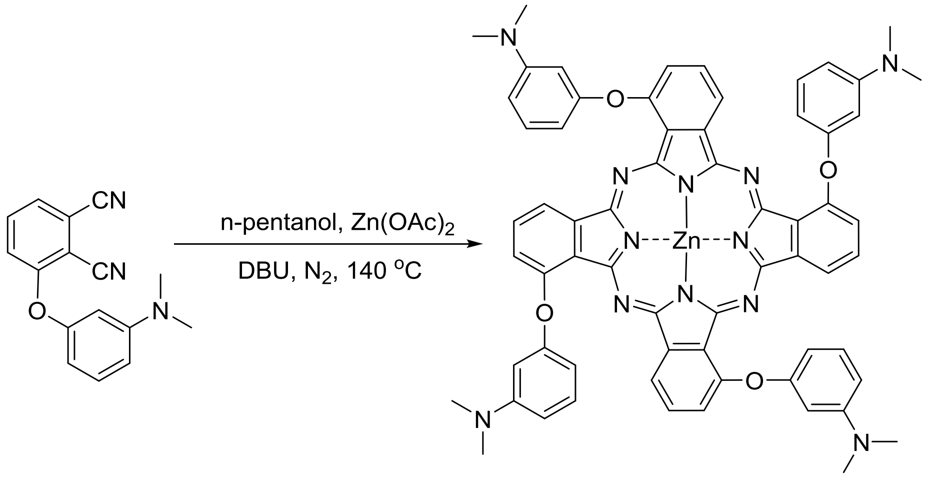

3-[3-(dimethylamino)phenoxy]phthalonitrile (7.6 mmol) in n-pentanol (40 mL) was stirred at 90 °C under an atmosphere of nitrogen for 30 min, and then zinc acetate (3.8 mmol) and 1,8-diazabicyclo-[5.4.0]undec-7-ene (DBU) (2 mL) were added. The resulting mixture was stirred at 140 °C for 48 h. After being cooled, the reaction mixture was poured into water (200 mL) and standed for 3 h. The green solid was filtered and washed with water and methanol, and then drying in vacuo. Then the crude product was first purified by silica gel column chromatography by using CH_2Cl_2 and EA as eluent to give a green crude. The mixture was

concentrated under reduced pressure and purified by size exclusion chromatography on a Bio-Beads S-X3 column using DMF as eluent. The crude product was further purified by recrystallization from a mixture of $CHCl_3$ and hexane to afford a green solid (1.01 g, 47.5%). ^{1}H NMR (300 MHz, $CDCl_3$): δ = 9.19-9.22 (m, 2 H, Pc-H), 8.72-9.11 (m, 2 H, Pc-H), 7.98-8.02 (m, 2 H, Pc-H), 7.75-7.83 (m, 2 H, Pc-H), 7.56-7.67 (m, 2 H, Pc-H), 7.34-7.49 (m, 2 H, Pc-H), 7.06-7.19 (m, 2 H, Ar-H), 6.64-6.89 (m, 6 H, Ar-H), 6.40-6.53 (m, 2 H, Ar-H), 6.21-6.32 (m, 4 H, Ar-H), 6.01-6.10 (m, 2 H, Ar-H), 2.52-2.78 (m, 12 H, -CH_3), 2.24-2.32 ppm (m, 12 H, -CH_3). HRMS (ESI): m/z Calcd for $C_{64}H_{53}N_{12}O_4Zn$ $[M+H]^+$ 1117.3599, found 1117.3463.

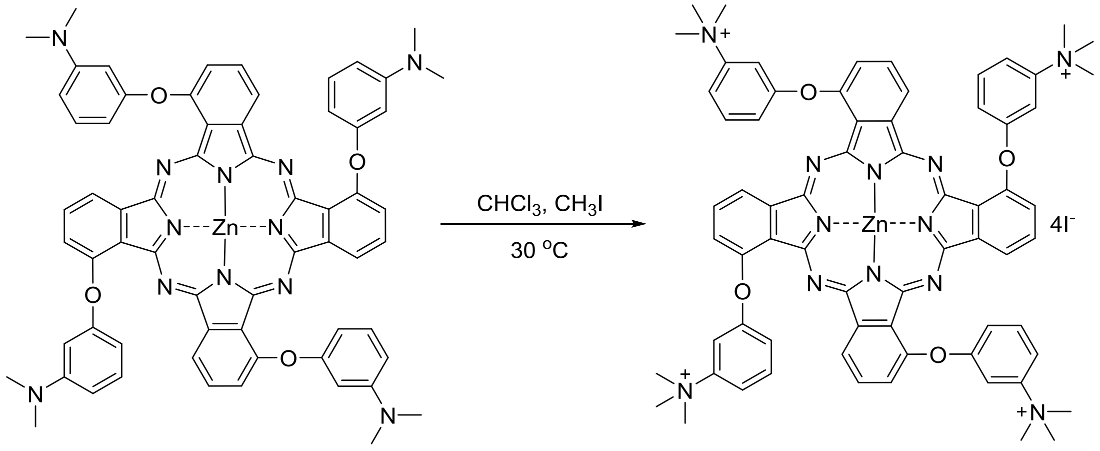

{1,8(11),15(18),22(25)-tetrakis[3-(dimethylamino)phenoxy]phthalocyaninate}zinc(II) (0.3 mmol) and CH_3I (11.4 mmol) were stired in $CHCl_3$ (20 mL) at room temperature for 48 h. Then the solid product was filtered and washed with $CHCl_3$, and then drying in vacuo. The product was afforded as a green solid $ZnPcN_4$ (0.4 g, 83 %). ^{1}H NMR (300 MHz, DMSO-d_6): δ = 9.47-9.52 (m, 2 H, Pc-H), 8.53-8.62 (m, 2 H, Pc-H), 8.40-8.44 (m, 2 H, Pc-H), 8.11-8.21 (m, 2 H, Pc-H), 7.95-8.04 (m, 4 H, Pc-H), 7.72-7.81 (m, 4 H, Ar-H), 7.19-7.57 (m, 10 H, Ar-H), 6.50-6.61 (m, 2 H, Ar-H), 3.79-3.85 (m, 21 H, -CH_3), 3.55-3.62 ppm (m, 15 H, -CH_3). HRMS (ESI): m/z Calcd for $C_{68}H_{64}N_{12}O_4Zn$ $[M-4I]^{4+}$ 294.1111, found 294.1109.
